# Atlantic salmon require long-chain *n*-3 fatty acids for optimal growth throughout the seawater period

**DOI:** 10.1017/jns.2016.10

**Published:** 2016-05-11

**Authors:** Grethe Rosenlund, Bente E. Torstensen, Ingunn Stubhaug, Nafiha Usman, Nini H. Sissener

**Affiliations:** 1 Skretting Aquaculture Research Centre, PO Box 48, 4001 Stavanger, Norway; 2 National Institute of Nutrition and Seafood Research (NIFES), PO Box 2029, Strandgaten 229, 5817 Bergen, Norway

**Keywords:** Atlantic salmon, *n*-3 Fatty acids, Fish requirements, Growth performance in seawater, BW, body weight, EFA, essential fatty acid, FA, fatty acid, FCR, feed conversion ratio, FO, fish oil, LC-PUFA, long-chain PUFA, LNA, *α*-linolenic acid, PL, phospholipid, SGR, specific growth rate, TGC, thermal growth coefficient, VO, vegetable oil

## Abstract

The nutritional requirement for *n*-3 long-chain PUFA in fast-growing Atlantic salmon (*Salmo salar*) during grow out in the sea is not well documented. Diets were formulated with levels of EPA (20 : 5*n-*3) and DHA (22 : 6*n*-3) ranging from 1·3 to 7·4 % of fatty acids (4–24 g/kg feed). Two long-term trials were conducted through the seawater phase, the first at 6 and 12°C, and the second at 12°C. In the first trial, growth at both temperatures was significantly lower in fish fed 1·4 % EPA+DHA of total fatty acids compared with the 5·2 % EPA+DHA group. In the second trial, growth was significantly lower in fish fed 1·3 and 2·7 % compared with 4·4 and 7·4 % EPA + DHA. Fatty acid composition in the fish reflected diet composition, but only after a 7-fold increase in body weight did the fatty acid profile of the fish stabilise according to dietary fatty acids (shown for EPA and DHA). The retention efficiency of DHA increased with decreasing dietary levels, and was 120–190 and 120–200 % in trials 1 and 2, respectively. The retention efficiency of EPA was lower (60–200 %), and values >100 % were only achieved at the lowest dietary levels in both trials. Temperature did not affect fatty acid retention efficiency. These results suggest that Atlantic salmon have a specific requirement for EPA + DHA >2·7 % of fatty acids for optimal long-term growth in seawater, and that short-term growth trials with less weight increase would not show these effects.

If fish oil (FO) inclusion in salmon feeds is to be kept at current levels, the supply of FO is the single factor that in the short term will limit further growth in production^(^^1^^)^. The exact requirement of Atlantic salmon (*Salmo salar*) for the long-chain PUFA (LC-PUFA) EPA (20 : 5*n-*3) and DHA (22 : 6*n*-3) is not known, but levels around 5–10 g/kg feed (1·5–3·0 % of lipids) have been suggested^(^^2^^,^^3^^)^. It is also possible that the requirements of salmon for these fatty acids (FA) at the tissue level could be fulfilled by dietary *α*-linolenic acid (18 : 3*n*-3, LNA), which then requires desaturation and elongation to EPA and DHA by the fish, mainly in the liver^(^^2^^)^. Adequate tissue levels of EPA and DHA are important to maintain fish health, as they play key roles in ontogenesis, growth, survival, pigmentation and resistance to stress and disease as well as in the development and functionality of the brain, vision and nervous system^(^^2^^)^.

In rainbow trout (*Oncorhynchus mykiss*), it was shown early on that *n*-3 FA (LNA and LC-PUFA) were essential for good growth and survival^(^^4^^,^^5^^)^. Diets with no fat or only oleic acid resulted in poor growth and clear deficiency symptoms such as fin damage, increased liver index, pale livers, histological changes in the heart and reduced survival. Some of these symptoms were partially alleviated by adding *n*-6 PUFA, while *n*-3 PUFA and LC-PUFA were demonstrated to be superior^(^^5^^)^. Including LNA or *n*-3 LC-PUFA in maize oil diets (high *n*-6 FA) resulted in increased growth and better survival^(^^4^^)^. Studies in Atlantic salmon in freshwater have shown that 10 g/kg feed as *n*-3 FA gave better growth, and fish fed a mixture of EPA and DHA grew better than fish fed only LNA^(^^6^^)^. All of the above studies were, however, conducted with semi-synthetic diets with low lipid content, resulting in poor growth, thus they are not directly applicable to the aquaculture industry today, with high-lipid diets and fast-growing fish. In view of these changes essential FA (EFA) requirements in important aquaculture species should be reassessed and in particular the understanding of the dietary needs for *n*-3 LC-PUFA must be improved^(^^7^^)^. Many studies have been conducted with Atlantic salmon in seawater replacing up to 100 % of the FO in the diets with vegetable oils (VO) in the grow-out stage in seawater^(^^8^^,^^9^^–^^20^^)^. However, the diets used in these studies contained fish meal as the main protein source, which also contains phospholipids (PL) rich in EPA and DHA, resulting in dietary levels of EPA + DHA at 10 g/kg and above. Hence, the EPA and DHA contents were not probably a limiting factor in these studies. Thus, there is a need for trials looking at the effects of low dietary EPA and DHA in fast-growing Atlantic salmon in the seawater stage assuring that weight gain is sufficient to minimise the effects of initial body levels of these FA.

During production in the sea, Atlantic salmon are exposed to seasonal variations in environmental temperature. Modification of FA composition in membrane PL is a major response to thermal change, and decreasing temperature will result in a greater proportion of PUFA in the membranes^(^^21^^–^^23^^)^. These changes are associated with changes in mitochondrial oxidative capacities. In particular, the content of EPA and DHA varies inversely with the content of 18 : 1*n*-9 and 18 : 2*n*-6 as temperature increases^(^^23^^)^. Hence, temperature may affect the requirement for EPA and DHA in Atlantic salmon for maintaining membrane functionality.

It has been shown that Atlantic salmon can be a net producer of DHA^(^^20^^)^. However, despite some *de novo* biosynthesis, tissue levels of DHA are reduced in fish fed VO compared with fish fed FO. Additionally, net production of DHA was only shown during a period with high growth and low DHA content in the diet^(^^20^^)^. Rainbow trout fed linseed oil can also be a net producer of EPA and DHA, as the amount recovered in fish was more than twice what the fish had been fed^(^^24^^)^. In salmonids fed linseed oil as the sole lipid source in the diet, liver FA desaturation and elongation activity was increased 2·6-fold, but tissue levels similar to fish fed FO could not be maintained^(^^25^^,^^26^^)^. Increased elongation and desaturation of LNA to EPA and DHA have been shown in hepatocytes isolated from Atlantic salmon fed EFA-deficient diets^(^^27^^)^.

The aim of the present study was to determine if low dietary levels of EPA and DHA, in the presence of LNA, affect the growth of Atlantic salmon in the seawater stage corresponding to >20-fold increase in body weight (BW). Furthermore, we aimed to elucidate if the requirement was affected by temperature, and also how the FA composition of whole fish and fillet, as well as the FA retention efficiency were affected by low dietary EPA and DHA contents.

## Materials and methods

### Feeding trials

Two long-term growth trials were conducted with Atlantic salmon (*Salmon salar* L.) in seawater using a regression design. In both trials all fish were individually tagged with micro-transponders (RFID tags; Trac Id Systems). For each pellet size and trial the experimental diets were produced from a common dry extruded feed kernel and differed only in the combination of oils added to the kernel. The diets were formulated to contain a gradient of EPA and DHA ranging from 1 to 5 % and from 1 to 7·5 % of total FA in trials 1 and 2, respectively (Tables 1 and 2, Supplementary Tables S1–S5). The main oil source added was a blend of plant oils (rapeseed–palm–linseed; 55:30:15)^(^^18^^)^ and the experimental gradient in EPA + DHA was obtained by including 0 to 35 % FO in the total oil mix added to the diets. Feeds were produced as 4, 6 and 8 mm pellets according to fish size. The fish meal level was kept constant at 10 % in all pellet sizes, but crude protein and lipid contents were formulated according to commercial salmon diets (Skretting, Norway), i.e. crude protein was formulated to 470, 430 and 380 g/kg and total lipid to 300, 320 and 340 g/kg in 4, 6 and 8 mm pellets, respectively.

**Table 1.** Composition and proximate composition (g/kg) of the experimental diets (8 mm) used in trial 1 from about 1500 g body weight (diets for trial 2 were formulated accordingly using the same raw materials)

Diet…	1:1·4	1:2·7	1:3·4	1:4·3	1:5·2
Ingredients					
Fishmeal*	100	100	100	100	100
Wheat gluten†	114	114	114	114	114
Sunflower-seed meal‡	40	40	40	40	40
Faba beans, dehulled§	60	60	60	60	60
Soya concentrate‖	275	275	275	275	275
Wheat§	50	50	50	50	50
Fish oil¶	0	22·4	43·5	64·6	85·7
Rapeseed oil§	175·0	161·9	150·3	138·7	127·1
Palm oil**	94·5	88·3	82·0	75·7	69·3
Linseed oil††	47·2	44·1	41·0	37·8	34·7
Premixes‡‡	44·30	44·30	44·30	44·30	44·30
Proximate composition					
Protein	395	385	388	388	389
Fat	355	359	356	348	352
Moisture	60	60	62	64	68

* Scandinavian fishmeal (Skretting).

† Cargill Cerestar.

‡ Linas Agro AS.

§ Skretting.

‖ Selecta.

¶ Northern hemisphere fish oil (Skretting).

** Palmolein, Fritex 24 (Aarhus Karlshamns).

†† Elbe Fetthandel GmbH.

‡‡ Include vitamins and minerals (Trouw Nutrition), proprietary composition Skretting ARC, vitamin and mineral supplementation as estimated to cover requirements according to the National Research Council^(^^3^^)^.

**Table 2.** Fatty acid composition (% of total FA) in the 8 mm diets used in trials 1 and 2

	Diet								
Fatty acids	1:1·4	1:2·7	1:3·4	1:4·3	1:5·2	2:1·3	2:2·7	2:4·4	2:7·4
Saturates									
14 : 0	0·6	1·2	1·6	1·9	2·4	0·7	1·1	1·7	3·2
16 : 0	14·8	14·8	14·9	15·0	15·1	15·1	14·9	15·0	14·5
18 : 0	2·3	2·2	2·1	2·2	2·2	2·5	2·3	2·6	2·6
Sum saturates	18·3	19·0	19·3	19·9	20·4	19·1	19·1	20·2	21·4
Monoenes									
16 : 1*n*-7/9	0·6	1·2	1·5	1·9	2·3	0·6	1·1	1·8	3·0
18 : 1*n*-9/7	42·8	42·4	39·1	37·7	35·6	42·7	40·7	37·8	31·8
20 : 1*n*-9	1·3	1·7	2·0	2·3	2·6	1·5	1·8	2·3	3·1
22 : 1*n*-11/9	0·8	1·4	1·8	2·2	2·6	1·1	1·4	2·2	3·7
Sum monoenes	45·9	45·3	45·0	44·7	43·7	46·4	45·5	44·5	42·2
*n*-6 Fatty acids									
18 : 2*n*-6	16·6	15·6	14·8	14·3	13·5	16·5	15·6	14·3	12·6
20 : 4*n*-6	0·0	0·1	0·1	0·1	0·2	0·0	0·1	0·2	0·3
Sum *n*-6	16·7	16·0	15·2	14·8	14·2	16·6	15·9	14·8	13·5
*n*-3 Fatty acids									
18 : 3*n*-3	13·2	12·0	11·5	10·7	10·1	11·7	11·1	10·1	9·0
18 : 4*n*-3	0·1	0·3	0·4	0·6	0·7	0·1	0·3	0·5	1·0
20 : 4*n*-3	0·0	0·1	0·1	0·1	0·2	0·0	0·1	0·1	0·3
20 : 5*n*-3	0·7	1·4	1·8	2·3	2·8	0·6	1·4	2·4	4·1
22 : 5*n*-3	0·1	0·2	0·2	0·3	0·4	0·1	0·2	0·3	0·5
22 : 6*n*-3	0·7	1·3	1·6	2·0	2·4	0·7	1·3	2·0	3·3
Sum *n*-3	14·8	15·4	15·7	16·1	16·7	13·2	14·5	15·5	18·4

**Fig. 1.**
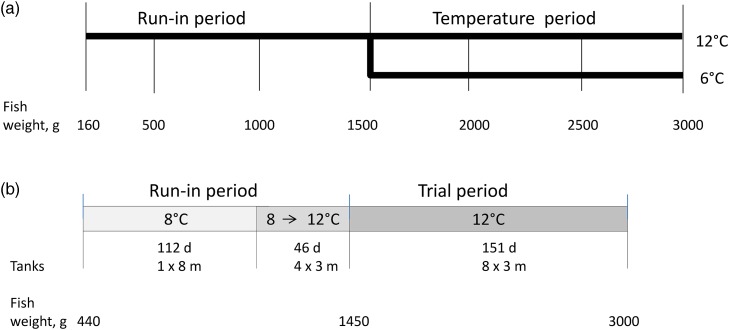
Outline of temperature regimens and corresponding developments in fish weight, in trial 1 (a) and trial 2 (b). In trial 1 fish were kept at 12°C during the run-in period of 216 d before tanks were split and continued at 12 and 6°C for 142 and 202 d, respectively. In trial 2 the run-in period consisted of 112 d at 8°C and 46 d of acclimatisation to experimental tanks and temperatures. The trial period lasted for 151 d at 12°C.

In trial 1, 750 Atlantic salmon, mean BW 162 (sem 0·9) g, were evenly distributed in five circular tanks (diameter 3 m, 7000 litres) supplied with flow-through seawater at 12°C. The photoperiod was 24 h light. Fish in each tank were fed one of the five experimental diets with graded levels of EPA + DHA from 1·4 to 5·2 % of total FA for a run-in period of 216 d (Fig. 1(a)). The biomass in each tank was then split into two new 3 m tanks and continued with the same diets. The temperature in one tank on each diet was continued at 12°C (11·8 (sem 0·02)°C), whereas the other during a 6 d period (Fig. 1(a)) was gradually decreased to 6°C (6·5 (sem 0·02)°C). At the start of the temperature trial the number of fish in each tank was 48–57, and the mean BW was 1442 (sem 19) g (*n* 264) and 1458 (sem 19) g (*n* 268) in the 12 and 6°C groups, respectively. The fish were fed the experimental diets (Table 1) until they reached an average BW of 3 kg (Fig. 1(a)), i.e. for 142 and 202 d at 12 and 6°C, respectively. Due to lower growth than expected at 12°C in trial 1 and a positive correlation between growth and dietary EPA + DHA content, it was decided to repeat this part with a wider range of dietary EPA + DHA levels.

In trial 2, Atlantic salmon (*n* 1200, BW 440 g) were fed a diet low in EPA + DHA (3 % of total FA, Supplementary Table S5) during a run-in period of 158 d to bring the level of EPA and DHA in the fish down to a common low start level. The fish were first kept in one 8 m tank (75 000 litres) with seawater at 8°C for 112 d before the fish were moved to four 3 m tanks and the water temperature gradually increased to 12°C to acclimatise the fish to the trial conditions (Fig. 1(b)). For the trial, a total of 560 fish with a mean BW of 1439 (sem 9) g were distributed equally in eight tanks (seventy fish per 3 m tank) supplied with flow-through seawater at 11·8 (sem 0·01)°C and photoperiod of 24 h light. Duplicate tanks (*n* 2) of Atlantic salmon were fed the experimental diets with graded levels of EPA + DHA from 1·3 to 7·4 % of total FA (Tables 1 and 2) for 151 d (Fig. 1(b)).

Both trials were performed at Skretting ARC Fish Trial Station (Lerang, Norway) and were conducted according to the guidelines of the Norwegian State Commission for Laboratory Animals, at an approved facility for research with animals and were approved by the responsible person for animal ethics at the facility. Diets were distributed in slight excess of expected feed intake using automatic feeders (Betten S1 mini; Betten Maskinstasjon). All tanks were equipped with waste feed collectors for the control of daily feed intake. Standard husbandry procedures at the research station were applied.

### Sampling and biometric indices

All experimental diets were sampled and stored at −20°C until analysis. Salmon were anaesthetised in a bath with tricaine methanesulfonate (FINQUEL MS-222, 7 g/l) before registration of RFID tag, BW and body length of all individual fish. Sampled fish were killed by an overdose of anaesthesia in a separate tank. Ten fish per tank were sampled for whole-body analyses of proximate and FA composition at the start and end of the temperature period in trial 1 whereas, in trial 2, ten fish were sampled from the start population and five fish from each tank at the end. The fish were opened and the weights of liver, gonad (if present) and gutted fish were registered before making pooled samples of the whole fish from each tank. Sexually mature fish were not included in the whole-body samples. In trial 1 individual fillet samples were collected at every 500 g growth interval for analyses of proximate and FA composition. Six fish were sampled from the start population and from each tank before the temperature period (about 1500 g BW, *n* 30). Eight fish per tank (*n* 80) were sampled at the end of the trial while three fish per tank were sampled at the intermediate sampling points (BW: 500, 1000, 2000 and 2500 g) for fillet composition. Similarly in trial 2, individual fillets were obtained from sixteen fish at the start and from four and six fish per tank at the intermediate and final samplings, respectively. Weights of liver, gonads and gutted fish were recorded for these fish. Condition factor was determined in all fish, and liver index (hepatosomatic index; %), gonadosomatic index (%) and dress-out percentage were determined in all sampled fish (>20 fish per tank) at the end of both trials. Liver was dissected from eight and six fish per tank at the end of trials 1 and 2, respectively. A small piece was immediately frozen in liquid N_2_ and stored at – 80°C for gene expression analysis.

### Proximate composition analyses

Diets, whole fish and fillets were analysed for content of DM, crude protein and total lipid using standard laboratory methods. DM and ash were measured gravimetrically after drying at 103°C for 24 h and after flame combustion at 550°C for 16–18 h, respectively^(^^28^^)^. Total N was determined using the Kjeldahl method and crude protein calculated as N × 6·25^(^^29^^)^. Total fat in feeds was measured gravimetrically after acid hydrolysis and extraction with diethyl ether^(^^30^^)^, whereas total fat in the fish samples was determined gravimetrically according to the AOCS^(^^31^^)^ after extraction with dichloromethane instead of petroleum ether in a Soxtec apparatus (Soxtec Auto Avanti).

### Fatty acid analysis

FA analysis was performed after methylation of the FA in methanolic HCl and extraction in hexane^(^^32^^)^. The FA composition was determined after separation of the methyl esters in a gas chromatograph (Thermo Trace GC with Triplus autosampler; Thermo Scientific), equipped with a cold on-column injector (90°C for 2 min, 30°C/min to 150°C, 3°C/min to 225°C, held for 5 min), a Varian 25-m CP Wax 52 capillary column (internal diameter 0·25 mm, film thickness 0·20 µm), a flame ionisation detector, and He as the carrier gas. The FA were identified by retention time using standard mixtures of methyl esters (Nu-Chek, Elyian), and the FA composition (percentage of total FA) was determined. All samples were integrated using the software Chromeleon^®^ version 6.8 connected to the GC. Amount of FA per g sample was calculated using 23 : 0 methyl-ester as internal standard. Data on whole-fish FA composition are presented as percentage of total FA, to facilitate comparison between samplings and temperatures when the fish differ in total lipid content. For retention calculations the mg/g data are used, as these were considered more reliable than combining the percentage of total FA data with lipid content determined gravimetrically, the latter being a relatively crude method with higher variability. The data as percentage of total FA are given in the Results section, while the mg/g data are provided as Supplementary material (Supplementary Tables S9–S11).

### Gene expression analyses

RNA purification, including homogenisation, DNase treatment and assessment of quantity and quality were done as described in Sissener *et al.*^(^^33^^)^. Average RNA integrity number for a selection of twelve samples in trial 1 was 8·2 (sd 0·2) and 9·5 (sd 0·2) at 12 and 6°C, respectively, and 9·1 (sd 0·5) in trial 2.

Reverse transcription was performed on a GeneAmp PCR 9700 (Applied Biosystems, AB) using the TaqMan® reverse transcriptase kit with oligo(dT) primers (Applied Biosystems). Primer sequences for Δ5- and Δ6-desaturase and the reference genes *β-actin* and *EF1α* have already been published elsewhere^(^^34^^,^^35^^)^. Samples were run in duplicate (500 ng, ± 5 %), in addition to a six-point dilution curve in triplicate (1000 to 31·25 ng), non-template and non-amplification controls. Real-time PCR amplification and analysis were performed on a LightCycler 480 Real-time PCR system (Roche Applied Science) with SYBR® Green I Mastermix (Roche Applied Science). Pipetting of plates was done using a Biomek®3000 Laboratory automation workstation (Beckman Coulter). Thermal cycling was done for forty-five cycles of 10 s at each of 95, 60 and 72°C (basic program from Roche), followed by melting analysis to confirm that only one product was present.

Cycle threshold (*C*_t_) values were calculated using the second maximum derivative method in the Lightcycler^®^ software. Amplification efficiency was determined using the dilution curves with the formula *E* = 10^(–1/slope), with the slope of the linear curve of *C*_*t*_ values plotted against the log-dilution^(^^36^^)^. Data analysis was conducted with GenEx 4.3.5 (MultiD Analyses AB), including efficiency correction, normalisation with both reference genes and averaging of technical replicates.

### Calculations


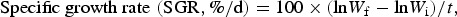
 where *W* is final and initial BW and *t* is number of days between *W*_i_ and *W*_f_. 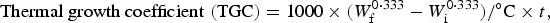 where *W* is final and initial BW, *t* is number of days between *W*_i_ and *W*_f_ and °C is mean temperature. 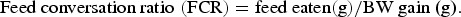


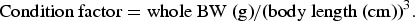


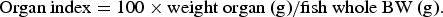


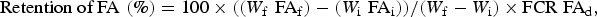
 where *W*_i_ and *W*_f_ are initial and final BW of individual fish, FA_i_, FA_f_ and FA_d_ are the initial and final FA content in mg/g in fish and diet, and FCR is FCR per tank.

### Statistics

For measuring statistical differences between the dietary groups for FA analysis, growth parameters and body indices, regression analysis was conducted on tank means (MATLAB and Statistics Toolbox Release 2013a; The MathWorks, Inc.) and one-way ANOVA was done on pseudoreplicates from the same tanks in trial 1 (*P* < 0·05) (MATLAB and Statistics Toolbox Release 2013a and GraphPad Prism5; GraphPad Software, Inc.), followed by *post hoc* Tukey's multiple comparison test. Hence, ANOVA results from trial 1 must be treated with caution due to the inability to distinguish between diet effects and tank effects in this statistical analysis. These results must be interpreted together with results from regression analysis and results from trial 2 to get the full picture of which effects are likely to be biologically meaningful. In trial 2, nested ANOVA was conducted on the performance data to account for potential tank effects (diet as fixed factor and tank as random factor). The parameters SGR, TGC and BW gain, which are often calculated on a tank basis in fish trials, were calculated for individual fish as these were pit tagged and we therefore had both initial and final BW for the same fish. Results are presented as means with their standard errors unless otherwise stated.

## Results

### Diets

Proximate composition was similar for all 8 mm diets (Table 1 and Supplementary Table S5). The analysed content of EPA + DHA in the diets in trial 1 was slightly higher than the formulated levels (1–5 %); 1·4, 2·7, 3·4, 4·4 and 5·2 % of total FA, respectively (Table 2). Analysed EPA + DHA levels in trial 2 diets were 1·3, 2·7, 4·4 and 7·4 % of total FA. For the remainder of the present article, the fish groups will be referred to by the number of the trial followed by the percentage of EPA + DHA in the diet, i.e. 1:1·4 refers to the fish in trial 1 fed 1·4 % EPA + DHA of total dietary FA.

### Fish performance

Mortality was low and independent of diet in both trials: 0·2 % during the temperature period in trial 1 and 0·4 % in trial 2. During the 216 d run-in period in trial 1, the fish grew from an average BW of 162 (sem 0·9) g (*n* 750) to 1433 (sem 13) g (*n* 649). The growth, expressed as gain and TGC, during this period was similar in diet groups 1:1·4 and 1:5·2 and slightly better in fish fed diets 1:2·7 and 1:3·4 with no linear regression (*P* = 0·97) to dietary EPA + DHA content (Table 3). Towards the end of the trial some fish started to develop gonads, resulting in reduced growth performance. This was confirmed using results for individual fish showing that all outliers in all groups were fish with low SGR (<0·35) and gonadosomatic index >1 % (data not shown). Thus, in order to get reliable results on dietary effects, further analyses were done using data from fish with SGR ≥0·35, which excluded fish with very low or negative growth in all groups. During the temperature period there were no significant differences in BW gain between groups at either temperature, but the regression between BW gain and dietary content of EPA + DHA was significant at 12°C (*R*^2^ 0·88, β 58, *P* = 0·02) (Table 3). Fish fed the diet with the highest EPA + DHA content (diet 1:5·2) had higher TGC (ANOVA, *P* < 0·05) than fish fed diet 1:1·4 at both temperatures (Table 3). Linear regression between TGC and dietary EPA + DHA content was significant at 12°C, but not at 6°C (Table 3).

**Table 3.** Performance and feed efficiency in Atlantic salmon fed dietary EPA + DHA from 1·4 to 5·2 % of total fatty acids for 142 and 202 d at 12 and 6°C, respectively† (Mean values with their standard errors)

	Diet 1:1·4		Diet 1:2·7		Diet 1:3·4		Diet 1:4·3		Diet 1:5·2			
	Mean	sem	Mean	sem	Mean	sem	Mean	sem	Mean	sem	Regression linear	ANOVA
Run-in period												
Initial weight (g)	161	2	163	2	161	2	163	2	163	2		NS
Final weight (g)	1360^a^	25	1480^b^	30	1478^b^	30	1467^a,b^	29	1379^a,b^	27	NS	**
Gain (g)	1201^a^	27	1320^b^	29	1315^b^	29	1302^a,b^	28	1207^a^	27	NS	**
TGC	2·16^a,b^	0·03	2·27^b^	0·03	2·21^b^	0·03	2·20^a,b^	0·03	2·16^a^	0·03	NS	**
12°C												
Initial weight (g)	1369	51	1518	47	1485	50	1476	56	1412	52	NS	NS
Final weight (g)	2780	132	3085	95	3056	115	3057	137	3074	124	NS	NS
Gain (g)	1410	87	1567	75	1571	70	1581	88	1662	80	*R*^2^ = 0·88, β = 58*	NS
Number of fish	32		40		33		34		41			
TGC	1·72^a^	0·07	1·80^a,b^	0·05	1·83^a,b^	0·04	1·84^a,b^	0·06	1·95^b^	0·06	*R*^2^ = 0·92, β = 0·055**	*
Feed intake‡ (g/fish)	1475		1502		1573		1599		1610			
FCR	1·02		0·98		1·04		1·02		0·93		NS	
6°C												
Initial weight (g)	1376^a,b^	54	1494^a,b^	48	1567^a^	58	1470^a,b^	62	1358^b^	44	NS	*
Final weight (g)	3249	121	3764	123	3833	165	3639	155	3556	134	NS	NS
Gain (g)	1873	76	2269	82	2266	113	2169	103	2198	95	NS	NS (*P* = 0·055)
Number of fish	27		34		32		34		44			
TGC	2·79^a^	0·06	3·11^b^	0·06	3·03^a,b^	0·08	3·03^a,b^	0·07	3·14^b^	0·07	NS	*
Feed intake‡ (g/fish)	2016		2135		2271		2082		1999			
FCR‡	1·05		0·96		0·98		0·98		0·91		NS	

TGC, thermal growth coefficient; FCR, feed conversion ratio.

^a,b^ Mean values within a row with unlike superscript letters were significantly different (*P* ≤ 0·05).

* *P* ≤ 0·05, ** *P* ≤ 0·01.

† Only fish with specific growth rate ≥0·35 are included in values for the temperature period using data from individually tagged fish. FCR values per tank during the temperature period. Prior to the temperature period the fish were fed the experimental diets during a run-in period of 216 d at 12°C.

‡ Calculated per tank including all fish.

The mean BW gain in trial 2 varied from 1804 (sem 51) g (*n* 120) in diet group 2:2·7 to 2151 (sem 56) g (*n* 120) in diet group 2:7·4 during the experimental period of 151 d (Table 4). BW gain in fish fed the two lowest dietary EPA + DHA contents (2:1·3 and 2:2·7) was significantly lower (nested ANOVA, *P* < 0·05) than in fish fed the diets with the highest EPA + DHA contents (2:4·4 and 2:7·4) (Table 4). The average TGC in trial 2 was 2·06 (sem 0·06) (*n* 8), and linear regressions were significant between dietary EPA + DHA content and BW gain (*P* = 0·02) and TGC (*P* = 0·02). As for mean BW gain, fish fed the diets with the two highest EPA + DHA contents had significantly higher (*P* < 0·05) TGC than fish fed the two lowest EPA + DHA levels (Table 4).

**Table 4.** Fish performance and feed efficiency in Atlantic salmon fed dietary EPA + DHA from 1·3 to 7·4 % of total fatty acids for 151 d at 12°C† (Mean values with their standard errors)

	Diet 2:1·3		Diet 2:2·7		Diet 2:4·4		Diet 2:7·4			
	Mean	sem	Mean	sem	Mean	sem	Mean	sem	Regression linear	Nested ANOVA
Initial weight (g)	1436	18	1447	18	1434	18	1434	18	NS	NS
Number of fish	140		140		139		139			
Final weight (g)	3252^a^	62	3243^a^	59	3420^a,b^	57	3597^b^	64	*R*^2^ = 0·56, β = 62*	*
Number of fish	122		120		117		120			
Gain (g)	1806^a^	54	1804^a^	51	2009^b^	45	2151^b^	56	*R*^2^ = 0·60, β = 62*	*
TGC	1·93^a^	0·05	1·94^a^	0·04	2·13^b^	0·04	2·23^b^	0·05	*R*^2^ = 0·66, β = 0·054*	*
FCR									*R*^2^ = 0·67, β = −0·014*	
Tank 1	1·16		1·16		1·08		1·05			
Tank 2	1·10		1·10		1·08		1·05			

TGC, thermal growth coefficient; FCR, feed conversion ratio.

^a,b^ Mean values within a row with unlike superscript letters were significantly different (*P* ≤ 0·05).

* *P* ≤ 0·05.

† Nested ANOVA was conducted using data from individually tagged fish followed by Tukey's honestly significant difference *post hoc* test and showed no significant differences between replicate tanks. Results are presented as mean values with their standard errors of individual fish per diet. FCR values are for each of the two tanks. The regression analyses were conducted on all eight tank means.

Fish growth in both trials was also assessed as relative growth index (RGI), based on Skretting's growth model (RGI calculator v7, Skretting; http://www.skretting.com/nb-NO/forskning--innovasjon/innovasjoner/aquasim1/), predicting growth as a function of fish size, temperature, feed intake, feed type and season. An RGI of 100 % in this model will be an average good growth under normal farming conditions of Atlantic salmon with normal health. The RGI for fish in trial 1 ranged from 110 to 122 % at 6°C and 77 to 88 % at 12°C, and from 90 to 100 % in trial 2. The decision to run the second trial was based on low RGI at 12°C in the first trial.

The FCR in trial 1 varied between 0·93 and 1·04 at 12°C and between 0·91 and 1·05 at 6°C (Table 3). At both temperatures, FCR was lowest for diet 1:5·2, but regression to dietary EPA + DHA content was not significant (*P* = 0·38 and *P* = 0·07 at 12 and 6°C, respectively). In trial 2, FCR was significantly reduced with increasing dietary contents of EPA + DHA (*R*^2^ 0·67; *P* = 0·03) (Table 4).

### Biometric indices

None of the biometric indices showed a significant dose–response relationship with the amount of EPA + DHA in the diet (*P* > 0·05) (Supplementary Tables S6 and S7).

### Fish proximate and fatty acid composition

**Fig. 2.**
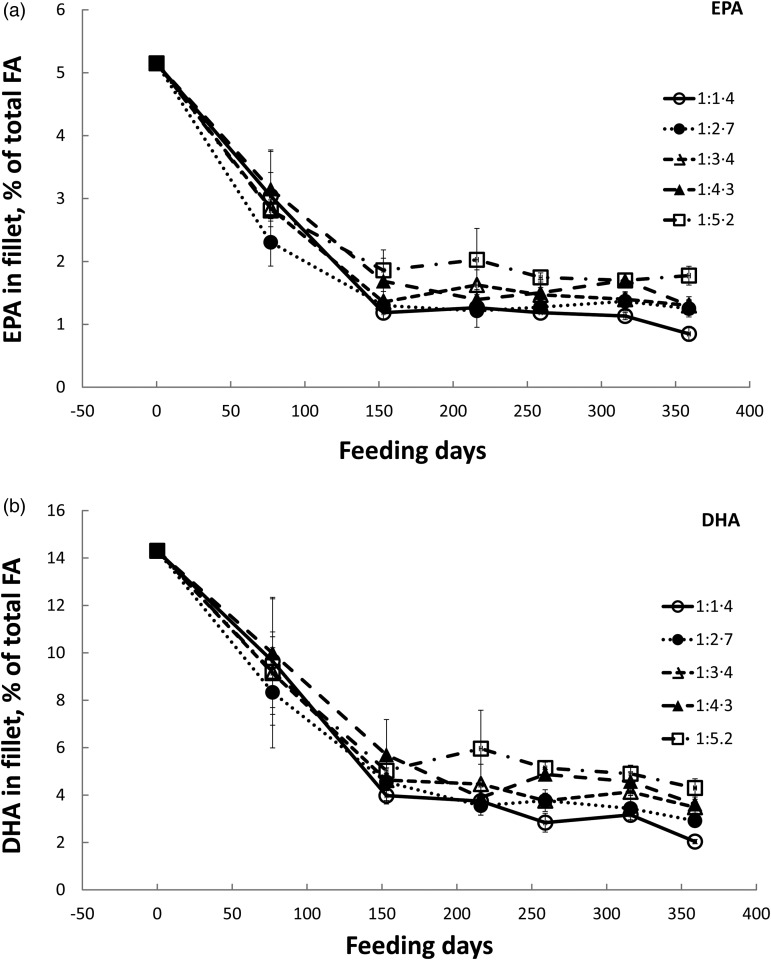
Changes in fillet content (% of total fatty acids (FA)) of EPA (a) and DHA (b) in Atlantic salmon fed dietary EPA + DHA levels from 1·4 to 5·2 % of total FA for 216 and 142 d at 12°C. Values are means of individual fish per tank (*n* 3–8), with standard deviations represented by vertical bars.

Proximate composition of the whole body in both trials was not affected by dietary EPA + DHA content (Supplementary Table S8); neither was fillet proximate composition (data not shown). The FA composition in fillet was determined at every 500 g growth interval in trial 1. As shown in Fig. 2, it took around 150 d before the fillet contents of EPA and DHA stabilised relative to diet. During this period the fish increased their BW nearly seven times. Whereas the content of EPA per diet group remained constant at 1·1 to 1·7 % of total FA during the temperature trial, DHA continued to decrease and ended at 2·0 to 4·2 % of total FA depending on dietary content, in diet groups 1:1·4 and 1:5·2, respectively. The diet-related changes in EPA and DHA content in fillet were not affected by temperature (Supplementary Table S9).

In trial 2, fillet content of EPA + DHA (percentage of total FA) was 4·8 % at the start of the trial (after the run-in period) and ended at 3·6, 4·1, 5·2 and 6·6 % in fish fed dietary levels of 1·3, 2·7, 4·4 and 7·4 % EPA + DHA of total FA (Supplementary Table S10). The FA composition in the whole body changed in a similar manner as the fillet composition during the trials (Tables 5 and 6). Both for EPA and DHA, there were gradients according to diet group, where fish receiving less in the diet also had a lower whole-body content. However, the differences in whole fish were smaller than the differences between the diets. In trial 1, DHA as percentage of total FA decreased in all diet groups during the temperature period, while EPA was more stable for each dietary group.

**Table 5.** Fatty acid composition (% of total fatty acids) in the whole body of Atlantic salmon at the start and end of the temperature period in trial 1†

	Start 12 and 6°C					Final at 12°C					Final at 6°C					Regression	
Diet…	1:1·4	1:2·7	1:3·4	1:4·3	1:5·2	1:1·4	1:2·7	1:3·4	1:4·3	1:5·2	1:1·4	1:2·7	1:3·4	1:4·3	1:5·2	12°C	6°C
Fatty acids																	
Saturates																	
14 : 0	1·0	1·3	1·5	1·7	2·0	0·7	1·1	1·3	1·6	1·9	0·7	1·1	1·4	1·6	1·9	****	***
16 : 0	13·2	13·6	13·5	13·4	13·1	12·5	12·9	13·1	12·9	13·2	12·1	12·3	12·5	12·4	12·5	NS	NS
18 : 0	3·1	3·2	3·0	3·0	2·8	3·2	3·1	3·1	3·0	3·0	3·1	3·1	3·1	2·7	2·9	**	NS
Total saturates	17·8	18·7	18·6	18·8	18·7	17·1	18·0	18·4	18·4	19·0	16·6	17·1	17·7	17·4	18·1	**	NS
Monoenes																	
16 : 1*n*-7/9	1·3	1·6	1·9	2·0	2·4	1·0	1·5	1·7	1·9	2·3	1·2	1·7	1·9	2·2	2·6	***	***
18 : 1*n*-9/7	41·8	39·5	38·7	38·7	36·6	42·8	41·1	40·1	38·6	37·3	44·1	42·7	41·1	39·9	38·2	****	***
20 : 1*n*-9	2·1	2·2	2·5	2·6	2·7	2·3	2·7	2·9	3·1	3·3	2·3	2·7	3·0	3·2	3·3	***	***
22 : 1*n*-11/9	0·9	1·1	1·3	1·5	1·7	0·8	1·2	1·5	1·7	2·1	0·7	1·1	1·4	1·6	2·0	***	*
Total monoenes	46·4	44·8	44·7	45·3	43·9	47·3	46·9	46·7	45·9	45·7	48·7	48·5	47·8	47·3	46·5	**	**
*n*-6 Fatty acids																	
18 : 2*n*-6	13·1	13·0	12·5	12·4	12·3	12·9	12·3	12·3	11·9	11·5	13·2	13·0	12·7	12·5	12·0	**	**
20 : 4*n*-6	0·4	0·3	0·3	0·3	0·3	0·2	0·2	0·2	0·2	0·2	0·3	0·2	0·2	0·2	0·2		NS
Total *n*-6	15·9	15·4	14·8	14·4	14·3	15·2	14·5	14·2	13·7	13·2	15·9	15·2	14·8	14·5	14·0	****	***
*n*-3 Fatty acids																	
18 : 3*n*-3	6·6	7·1	6·8	6·6	6·9	6·9	6·8	7·0	6·8	6·9	6·7	6·9	7·0	7·1	6·8	NS	NS
18 : 4*n*-3	2·2	1·6	1·5	1·5	1·3	1·8	1·3	1·3	1·2	1·1	2·0	1·5	1·3	1·1	1·1	*	**
20 : 4*n*-3	0·8	0·8	0·8	0·8	0·8	0·8	0·8	0·7	0·8	0·8	0·9	0·9	0·9	0·9	0·9	NS	
20 : 5*n*-3	1·3	1·5	1·5	1·7	2·1	1·0	1·2	1·3	1·4	1·7	1·1	1·3	1·4	1·7	1·9	**	**
22 : 5*n*-3	0·5	0·6	0·6	0·7	0·8	0·4	0·5	0·5	0·6	0·7	0·5	0·6	0·6	0·7	0·8	**	**
22 : 6*n*-3	3·4	3·9	4·5	4·6	5·2	2·1	2·5	3·0	3·4	3·9	2·0	2·3	2·7	3·3	3·9	***	**
Total *n*-3	15·1	15·9	16·1	16·4	17·5	13·2	13·5	14·3	14·7	15·6	13·3	13·9	14·4	15·3	15·9	**	***

* *P* ≤ 0·05, ** *P* ≤ 0·01, *** *P* ≤ 0·001, **** *P* ≤ 0·0001.

† Values represent pooled samples of ten fish per dietary treatment.

**Table 6.** Fatty acid composition (% of total fatty acids) in the whole body of Atlantic salmon at the start and end of trial 2†

			Final								
Diet…	Initial		2:1·3		2:2·7		2:4·4		2:7·4		
	Mean	sem	Tank 1	Tank 2	Tank 1	Tank 2	Tank 1	Tank 2	Tank 1	Tank 2	Regression
Fatty acids											
Saturates											
14 : 0	1·3	0·0	1·0	0·9	1·3	1·2	1·5	1·5	2·4	2·4	****
16 : 0	12·7	0·1	13·2	13·1	13·3	13·1	13·2	13·3	13·3	13·1	NS
18 : 0	3·1	0·1	2·9	2·8	2·8	2·8	2·9	2·8	2·8	2·8	NS
Total saturates	18·0	0·2	17·8	17·5	18·2	17·8	18·5	18·4	19·4	19·2	****
Monoenes											
16 : 1*n*-7/9	1·6	0·0	1·1	1·15	1·4	1·5	1·8	1·8	2·5	2·5	****
18 : 1*n*-9/7	40·4	0·1	41·7	42·0	40·5	41·1	39·0	39·4	35·4	35·8	****
20 : 1*n*-9	2·8	0·0	2·2	2·1	2·5	2·6	2·8	2·9	3·15	3·2	****
22 : 1*n*-11/9	1·5	0·1	0·9	0·9	1·2	1·2	1·6	1·5	2·2	2·2	****
Total monoenes	47·1	0·2	46·4	46·9	46·2	46·9	45·9	46·3	44·0	44·2	***
*n*-6 Fatty acids											
18 : 2*n*-6	13·3	0·1	14·2	14·1	13·8	13·6	13·1	12·95	12·2	12·2	****
20 : 4*n*-6	0·2	0·0	0·2	0·2	0·2	0·2	0·2	0·2	0·2	0·2	**
Total *n*-6	15·4	0·1	16·8	16·5	16·1	15·8	15·1	15·1	14·2	14·2	****
*n*-3 Fatty acids											
18 : 3*n*-3	7·0	0·1	7·7	7·8	8·0	7·7	7·7	7·4	7·4	7·4	*
18 : 4*n*-3	1·2	0·1	1·8	1·7	1·5	1·3	1·1	1·2	1·2	1·2	*
20 : 4*n*-3	0·9	0·1	1·0	0·8	0·9	0·9	0·9	0·8	1·0	1·0	NS
20 : 5*n*-3	1·5	0·1	1·3	1·3	1·6	1·5	1·8	1·7	2·5	2·4	****
22 : 5*n*-3	0·6	0·0	0·4	0·4	0·5	0·5	0·6	0·6	0·9	0·9	****
22 : 6*n*-3	2·9	0·1	2·4	1·95	2·8	2·4	3·3	3·1	4·2	4·3	***
Total *n*-3	14·7	0·1	15·1	14·5	15·9	14·9	16·1	15·5	17·9	18·0	***

* *P* ≤ 0·05, ** *P* ≤ 0·01, *** *P* ≤ 0·001, **** *P* ≤ 0·0001.

† Values represent pooled samples of five fish per tank at final sampling and mean values with their standard errors for four pooled samples of five fish each at initial sampling.

### Fatty acid retention

**Fig. 3.**
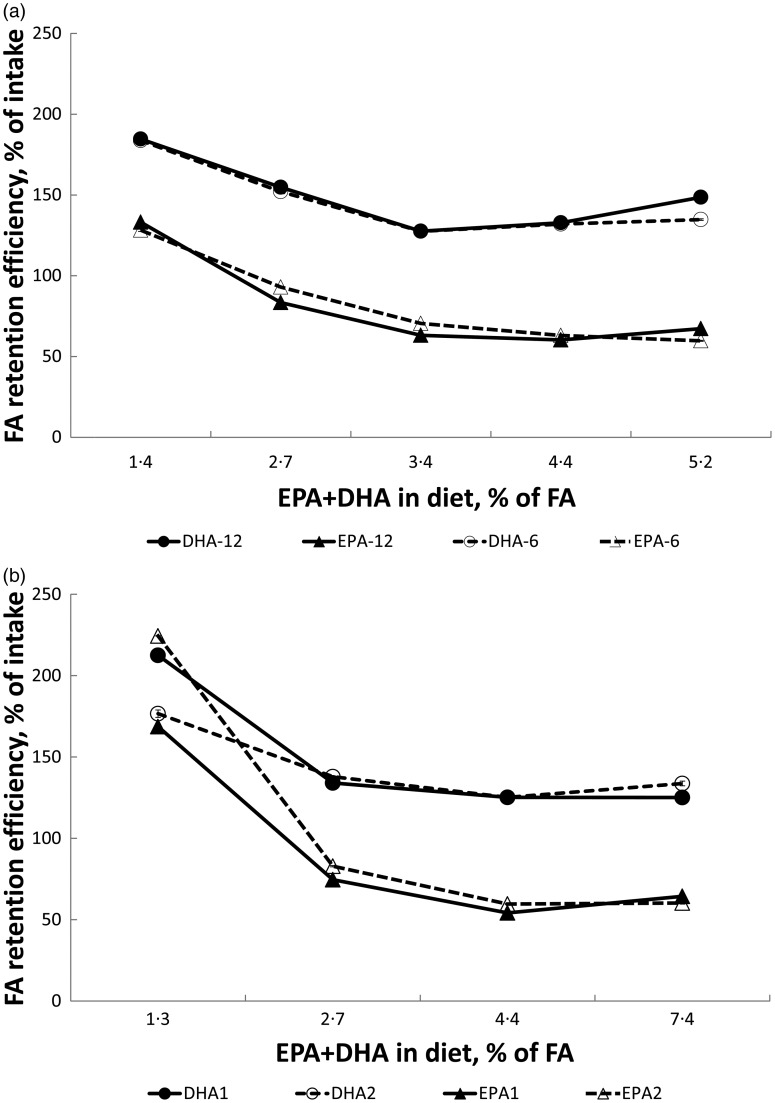
Retention efficiency of EPA and DHA in Atlantic salmon fed dietary EPA + DHA levels from 1·4 to 5·2 % of total fatty acids (FA) for 202 and 142 d at 6 and 12°C, respectively (a) and 1·3 to 7·4 % of total FA for 151 d at 12°C (b). Values are means per tank, with standard errors represented by vertical bars.

Retention efficiency of dietary EPA and DHA was calculated using BW gain values for individual fish, FA contents in feeds and whole body expressed as mg/g (Supplementary Tables S11–S13) and estimated feed intake using FCR per tank. In both trials, net production (retention efficiency values >100 %) of DHA was found in all diet groups (Fig. 3). However, the DHA retention efficiency was fairly constant when dietary levels of EPA + DHA were 3–4 % of total FA and above, but increased sharply when dietary EPA + DHA levels decreased, reaching around 200 % at the lowest dietary level. A similar response was also seen for EPA, but in contrast to DHA, net production of EPA was only observed at the lowest EPA + DHA level. Temperature did not seem to affect retention efficiency of EPA and DHA in Atlantic salmon.

### Gene expression

Significant linear regressions were seen both for Δ5-desaturase (*R*^2^ 0·94, β −0·97, *P* = 0·004) and Δ6-desaturase (*R*^2^ 0·80, β −0·92, *P* = 0·025) at 6°C, with expression decreasing with increasing dietary EPA + DHA (Supplementary Fig. S1). At 12°C, despite a similar trend, linear regressions were not significant for the same genes, possibly due to high variation and high expression in some individuals in diet group 1:4·3. In trial 2, fish in diet groups 2:1·3 and 2:2·7 appeared to have higher expression of these two genes than fish in diet groups 1:4·4 and 2:7·4. However, there was large individual variation and these differences were not significant.

## Discussion

EFA requirements in salmonids have been examined previously^(^^4^^–^^6^^)^. However, all these trials were performed in the freshwater stage, with low-fat diets and poor growth compared with common growth in commercial production. Consequently, EPA + DHA requirements need to be reassessed in light of current production diets for salmonids, which have high levels of lipids and energy to support high growth rates^(^^7^^)^. The present trial used diets that were as close to a commercial high-energy diet as possible, while EPA + DHA should be the limiting factor in the diet. As 18 : 3*n*-3 is present in most oil sources relevant for replacement of FO in commercial aquaculture, we chose to investigate the EPA + DHA requirement in the presence of dietary 18 : 3*n*-3. The VO mix used in this trial has not affected fish growth in previous trials^(^^18^^,^^37^^)^, and the observed effects can therefore be explained by the gradients in dietary EPA + DHA levels. There are indications that, at least for some species, EFA requirements might depend on lipid inclusion and hence be better expressed as percentage of the dietary FA^(^^38^^,^^39^^)^, which is done in this discussion.

The results from both trials and at both temperatures suggest a requirement for optimal growth between 2·7 and 3·4 % EPA + DHA of total FA. The fact that salmon perform better with dietary EPA + DHA compared with short-chain *n*-3 (LNA) has also been shown previously in the freshwater phase^(^^6^^)^. Few long-term trials in seawater have been conducted with salmon fed low EPA + DHA; however, in one trial of about 13-month duration in which EPA + DHA constituted only 2·7 % of total FA in a VO diet, there was reduced growth compared with a FO diet in two out of three salmon strains tested^(^^40^^)^. These data support our finding of >2·7 % EPA + DHA being required for optimal long-term growth in seawater, but also suggest family differences in the ability to cope with low dietary EPA + DHA^(^^40^^)^. This could be related to family differences in the flesh content of EPA + DHA of fish fed the same diets^(^^41^^)^, and supports the usefulness of genetic selection for such traits. Another study feeding low EPA + DHA to post-smolt salmon was probably of too short duration to reveal significant growth differences, but noticeably fish fed 0·9 or 2·6 % EPA + DHA of total FA had (non-significantly) lower final BW than fish fed 4·1 % or more^(^^42^^)^, correlating well with our results. The exact pathways affected by EFA deficiency that reduce growth in animals have not been determined^(^^7^^)^.

The fact that the dietary effect on growth in trial 1 only became apparent in the temperature period (about 1500–3000 g), and not in the run-in period (about 160–1500 g), clearly shows that long-term trials are necessary when determining FA requirements for optimal growth. A 9-fold increase in BW was achieved in the run-in period, and most feeding trials conducted with Atlantic salmon are shorter than this, even when effects of EFA have been studied^(^^6^^,^^42^^)^. A 7-fold increase in BW was required for muscle FA profile to stabilise relative to the diets, which proves the importance of feeding diets low in EPA + DHA for a long period of time to deplete body stores. For whole fish and muscle in salmon, there will be a high proportion of storage lipids (TAG) relative to PL, the latter being central components of cell membranes. While incorporation of dietary FA in TAG generally follows a dilution model, incorporation in PL is much more complex^(^^43^^)^, for instance in salmon fed an EFA-deficient diet, EFA in liver PL seemed to be conserved at the expense of TAG^(^^44^^)^. The FA composition of tissues does not only reflect diet, but is modified by processes such as preferential incorporation, β-oxidation, lipogenic activity and elongation and desaturation of FA^(^^45^^)^. Consequently, the FA profile in tissues other than muscle may take even longer to stabilise after a diet change. In Atlantic salmon fed a diet completely deficient of all *n*-3 and *n*-6 FA, symptoms of deficiency only appeared in the fourth month of the study, while severe EFA deficiency did not develop^(^^44^^)^, further supporting the need for long-term trials.

Differences between fish reared at the two temperatures in SFA, MUFA and *n*-6 FA may indicate some thermal adaptation; however, no temperature effects were seen in EPA, DHA or total *n*-3 content in whole fish. If the main changes occur in cell membranes, these differences in the PL would probably be masked by the FA composition of the TAG which is present in much higher concentration than PL in whole fish and muscle. Hence, analyses of more PL-rich tissues could be more useful in this regard, which has been done by Sissener *et al.*^(^^46^^)^ using samples from these long-term trials. A study documenting *n*-3 content during the whole seawater production cycle in salmon fed the same feed in fish farms along the Norwegian coast concluded that temperature did not affect most FA, but had a slight effect on DHA which decreased in muscle with increasing temperature^(^^47^^)^. An effect of temperature on DHA in muscle was not seen in our study. However, that study was conducted with dietary EPA + DHA at about 18–19 % of total FA, thus providing these FA in large excess of requirements, unlike the present study.

The very similar results seen for retention efficiency between the two temperatures in trial 1 and between the trials firmly support that net production (retention values >100 %) of DHA occurs for all diet groups but increasingly so at the lowest dietary levels, and that DHA is retained to a higher degree than EPA. Higher retention for DHA than EPA is supported by previous feeding trials in Atlantic salmon^(^^19^^,^^20^^)^ and by a study on salmon hepatocytes^(^^48^^)^. Furthermore, many trials have observed that when DHA is reduced in the diet, the reduction observed in fish tissues is much lower than in the dietary lipids^(^^11^^)^, and that DHA is selectively deposited in muscle lipids regardless of concentration in the diet, while EPA and all other FA are increasingly used for metabolism as their dietary concentrations increase^(^^15^^)^. All this highlights the important structural role of DHA in salmonids, however, not disregarding the functional importance of EPA.

The retention efficiency of DHA did not seem to increase linearly with decreasing dietary content; however, there seemed to be a sharp increase in retention efficiency (close to 200 % in both trials) at dietary levels below what was needed to sustain optimal growth. A sharp increase in DHA retention efficiency when diet levels are deficient has also been observed by others^(^^42^^)^. Reduced retention efficiency at increased dietary levels seems to be due to feedback inhibition by *n*-3 long-chain FA^(^^49^^)^, probably DHA rather than EPA^(^^48^^)^. Regulation of synthesis of long-chain *n*-3 FA in Atlantic salmon involves the modulation of gene expression of fatty acyl desaturases^(^^35^^)^. This was also observed in our experiment, with tendencies towards increased expression of both Δ5- and Δ6-desaturase with increasing retention efficiency in the groups fed low dietary EPA + DHA. Effects of diet on metabolic genes are often subtle but still physiologically relevant^(^^50^^)^, which also seemed to be the case in this study. Our data showed no temperature difference in retention of EPA or DHA, while others^(^^51^^)^ observed a non-significant tendency of increased *n*-3 retention at lower temperature (2 *v*. 8°C) in salmon parr. However, only retention of total *n*-3 FA was calculated in that study and the experiment was conducted in the freshwater stage, making direct comparisons with our data difficult.

At the dietary levels where the EPA + DHA requirement for optimal growth was fulfilled (>2·7 % of FA), retention of these two FA together are around 100 %. Hence, salmon aquaculture would not need to diminish the world supply of these FA. However, using salmon for ‘production’ of long-chain *n*-3 FA does not seem like a realistic scenario. Furthermore, factors such as lower growth or increased economic FCR due to increased feed waste or mortalities in commercial farming compared with our experimental conditions will probably reduce the retention efficiency to less than 100 %.

Our results show that salmon will remain a good source of EPA and DHA for human nutrition also in the future. A 200 g portion of fillet of salmon fed diet 1:3·4 (fulfilling the requirements for optimal growth of the fish) in this study would provide about 1·25 g EPA + DHA (assuming an average fat content of 16 % in the commercial product), which is five times the daily dose of 250 mg to prevent the development of CVD in healthy adults recommended by the European Food Safety Authority^(^^52^^)^, while other recommendations are higher reviewed by VKM^(^^53^^)^.

### Conclusions

Atlantic salmon have a specific requirement for EPA + DHA for optimal growth in the sea and the required level is >2·7 % of FA (10 g/kg feed DM in a typical salmon grower diet) equally at 6 and 12°C. Our studies demonstrate that long-term trials are needed when FA requirement studies are carried out in post smolt salmon. Furthermore, salmon can be a net producer of DHA dependent on dietary supply, but retention increases significantly only when dietary levels drop below those required for optimal growth. Temperature (6 or 12°C) did not seem to have a major impact on the results obtained in the present study.

## Supplementary material

For supplementary material accompanying this paper visit http://dx.doi.org/10.1017/jns.2016.10. click here to view supplementary material
