# Supplementary material for: Atlantic salmon require long-chain n-3 fatty acids for optimal growth throughout the seawater period
Source: J Nutr Sci. 2016 May 11;5:e19. doi: 10.1017/jns.2016.10 (PMC4891698; doi:10.1017/jns.2016.10)
Supplement: Supplementary file 1 [file S2048679016000100sup001.pdf]

Table S1. Composition and proximate composition (g kg^{-1}) of the experimental diets (4 mm) used in trial 1 from start of run-in period to ~500 g body weight.

**Diet**	**1:1.4**	**1:2.7**	**1:3.4**	**1:4.3**	**1:5.2**
**Ingredients**					
Fish meal[†]	100	100	100	100	100
Wheat gluten[‡]	200	200	200	200	200
Faba beans, dehulled[§]	20.3	20.3	20.3	20.3	20.3
Soya concentrate[ǀ]	296.2	296.2	296.2	296.2	296.2
Wheat[§]	50	50	50	50	50
Fish oil[ǀ]	0	17.3	34.6	51.8	69.0
Rapeseed oil[§]	154	144.5	135	125.5	116.1
Palm oil[¶]	84	78.8	73.6	68.5	63.3
Linseed oil[††]	42	39.4	36.8	34.2	31.7
Premixes[‡‡]	53.5	53.5	53.5	53.5	53.5
**Proximate composition**					
Protein	476	474	461	462	463
Fat	305	301	299	309	294
Moisture	51	55	57	57	57

[†]Scandinavian fish meal, Skretting, Stavanger, Norway, [‡]Cargill Cerestar, Hautbourdin, France, [§]Skretting, Stavanger, Norway, [ǀ]Selecta, Goiâna-GO, Brazil, [ǀ]Northern hemisphere fish oil, Skretting, Stavanger, Norway, [¶]Palmolein, Fritex 24, Aarhus Karlshamns, Karlshamn, Sweden, [††]Elbe Fetthandel GmbH, Geesthacht, Germany, [‡‡]Include vitamins, minerals, methionine and lysine; Trouw Nutrition, Boxmeer, the Netherlands, proprietary composition Skretting ARC, vitamin and mineral supplementation as estimated to cover requirements according NRC (2011)

Table S2. Composition and proximate composition (g kg^{-1}) of the experimental diets (6 mm) used in trial 1 from 500 to 1000 g body weight.

**Diet**	**1:1.4**	**1:2.7**	**1:3.4**	**1:4.3**	**1:5.2**
**Ingredients**					
Fish meal[†]	100	100	100	100	100
Wheat gluten[‡]	160	160	160	160	160
Sunflower meal[§]	20	20	20	20	20
Faba beans, dehulled[ǀ]	40	40	40	40	40
Soya concentrate[ǀ]	275.5	275.5	275.5	275.5	275.5
Wheat[ǀ]	50	50	50	50	50
Fish oil[¶]	0	19.3	37.5	55.8	74.1
Rapeseed oil[ǀ]	165	154.4	144.3	134.3	124.3
Palm oil[††]	90	84.2	78.7	73.3	67.8
Linseed oil[‡‡]	45	42.1	39.4	36.6	33.9
Premixes[§§]	54.5	54.5	54.5	54.5	54.5
**Proximate composition**					
Protein	430	445	439	423	418
Fat	324	305	314	323	335
Moisture	59	52	55	59	61

[†] Scandinavian fish meal, Skretting, Stavanger, Norway, [‡]Cargill Cerestar, Hautbourdin, France,[§]Linas Agro AS, Aalborg, Denmark, [ǀ]Skretting, Stavanger, Norway, [ǀ]Selecta, Goiâna-GO, Brazil, [¶]Northern hemisphere fish oil, Skretting, Stavanger, Norway, [††]Palmolein, Fritex 24, Aarhus Karlshamns, Karlshamn, Sweden, [‡‡]Elbe Fetthandel GmbH, Geesthacht, Germany, [§§]Include vitamins, minerals, methionine and lysine; Trouw Nutrition, Boxmeer, the Netherlands, proprietary composition Skretting ARC, vitamin and mineral supplementation as estimated to cover requirements according NRC (2011)

Table S3. Fatty acid composition (% of total FA) of the 4 mm diets used in trial 1.

**Diet**	**1:1.4**	**1:2.7**	**1:3.4**	**1:4.3**	**1:5.2**
Fatty acid					
14:0	0.78	1.08	1.41	1.71	2.01
16:0	15.24	15.13	15.20	15.20	15.43
18:0	3.05	2.96	2.91	2.87	2.87
Sum saturates	*20.19*	*20.25*	*20.58*	*2083*	*21.30*
16:1*n*-7/9	0.74	1.04	1.37	1.66	1.91
18:1*n*-9/7	42.04	39.97	38.54	38.86	35.50
20:1*n*-9	1.22	1.42	1.61	1.87	2.10
22:1*n*-11/9	0.71	1.11	1.50	1.95	2.38
Sum monoenes	*45.08*	*44.05*	*43.59*	*42.91*	*42.40*
18:2*n*-6	16.64	16.13	15.31	14.99	14.62
20:4*n*-6	0.05	0.08	0.12	0.14	0.20
Sum n-6	*16.96*	*16.56*	*15.84*	*15.64*	*15.35*
18:3*n*-3	10.82	10.18	9.68	9.08	8.69
18:4*n*-3	0.26	0.41	0.54	0.66	0.80
20:4*n*-3	0.05	0.08	0.12	0.14	0.20
20:5*n*-3	1.31	1.68	2.13	2.62	3.16
22:5n-3	0.20	0.33	0.30	0.36	0.42
22:6*n*-3	1.02	1.49	1.90	2.41	2.99
Sum n-3	*13.72*	*14.28*	*14.81*	*15.46*	*16.46*

Table S4. Fatty acid composition (% of total FA) of the 6 mm diets used in trial 1.

**Diet**	**1:1.4**	**1:2.7**	**1:3.4**	**1:4.3**	**1:5.2**
Fatty acid					
14:0	0.74	1.12	1.37	1.65	2.01
16:0	15.29	15.24	15.34	15.16	14.27
18:0	2.46	2.41	2.39	2.38	2.23
Sum saturates	*19.04*	*19.32*	*19.68*	*19.72*	*19.03*
16:1*n*-7/9	0.74	1.12	1.36	1.61	1.95
18:1*n*-9/7	43.11	41.03	39.80	38.95	37.73
20:1*n*-9	1.55	1.68	1.91	2.05	2.28
22:1*n*-11/9	0.72	1.12	1.33	1.64	1.93
Sum monoenes	*46.73*	*45.62*	*45.11*	*44.97*	*44.76*
18:2*n*-6	16.29	16.01	15.20	14.56	14.33
20:4*n*-6	0.00	0.07	0.10	0.12	0.15
Sum n-6	*16.58*	*16.51*	*15.76*	*15.16*	*15.08*
18:3*n*-3	10.93	10.22	9.83	9.30	9.18
18:4*n*-3	0.28	0.46	0.52	0.62	0.75
20:4*n*-3	0.00	0.07	0.10	0.12	0.16
20:5*n*-3	0.73	1.36	1.74	2.13	2.67
22:5n-3	0.13	0.18	0.25	0.28	0.35
22:6*n*-3	0.75	1.39	1.77	2.04	2.54
Sum n-3	*12.82*	*13.78*	*14.32*	*14.61*	*15.79*

Table S5. Composition and proximate composition (g kg^{-1}) of the experimental diets used in trial 2.

**Diet**	**Run-in diet**	**2:1.3**	**2:2.7**	**2:4.4**	**2:7.4**
**Ingredients**					
Fish meal[†]	100	100	100	100	100
Wheat gluten[‡]	170	101.9	101.9	101.9	101.9
Sunflower meal[§]	20	40	40	40	40
Faba beans, dehulled[|]	40	60	60	60	60
Soya concentrate[|]	294.5	275	275	275	275
Wheat[|]	50	50	50	50	50
Fish oil[¶]	9.6	0	31.0	66.5	110.9
Rapeseed oil[|]	154.3	175.0	156.8	137.2	112.8
Palm oil[††]	84.2	94.0	85.5	74.9	61.5
Linseed oil[‡‡]	42.1	47.0	42.8	37.4	30.8
Premixes[§§]	35.3	57.1	57.1	57.1	57.1
**Proximate composition**					
Protein	431	382	383	386	382
Fat	324	337	343	347	331
Moisture	63	74	67	68	69

[†] Scandinavian fish meal, Skretting, Stavanger, Norway, [‡]Cargill Cerestar, Hautbourdin, France,[§]Linas Agro AS, Aalborg, Denmark, [|]Skretting, Stavanger, Norway, [|]Selecta, Goiâna-GO, Brazil, [¶]Northern hemisphere fish oil, Skretting, Stavanger, Norway, [††]Palmolein, Fritex 24, Aarhus Karlshamns, Karlshamn, Sweden, [‡‡]Elbe Fetthandel GmbH, Geesthacht, Germany, [§§]Include vitamins, minerals, methionine and lysine; Trouw Nutrition, Boxmeer, the Netherlands, proprietary composition Skretting ARC, vitamin and mineral supplementation as estimated to cover requirements according NRC (2011)

Table S6. Condition factor (CF), liver index (HSI, %), gonadosomatic index (GSI, %) and dress-out percentage (DO%) in Atlantic salmon fed dietary EPA+DHA from 1.4 to 5.2 % of total FA for 216 days at 12°C followed by 142 and 202 days at 12 and 6°C, respectively. Mean values with their standard error of individual fish with SGR $\geq$ 0.35 per tank. Values within a row with common superscript letters are not significantly different. *P*-values $\leq$ 0.05 are indicated by *, *p*-values $\leq$ 0.01 by ** and *p*-values $\leq$ 0.001 by ***.

	Fish/tank	Diet 1:1.4		Diet 1:2.7		Diet 1:3.4		Diet 1:4.3		Diet 1:5.2			*P* (One way
	n	Mean	SEM	Mean	SEM	Mean	SEM	Mean	SEM	Mean	SEM	*P* (regression)	ANOVA)
12°C													
CF	32-41	1.57	0.02	1.51	0.01	1.57	0.01	1.61	0.03	1.58	0.02	NS	NS ($p = 0.054$)
HSI	26-40	1.04	0.03	1.05	0.02	1.02	0.02	1.06	0.03	1.05	0.03	NS	NS
GSI	13-21	0.22^{a}	0.04	0.35^{ab}	0.04	0.41^{b}	0.05	0.38^{ab}	0.06	0.24^{a}	0.02	NS	**
DO%	26-40	87.5	0.4	88.6	0.2	88.6	0.3	88.6	0.2	87.8	0.4	NS	NS
6°C													
CF	27-43	1.76^{a}	0.03	1.73^{ab}	0.02	1.79^{a}	0.03	1.66^{b}	0.02	1.71^{ab}	0.02	NS	**
HSI	22-27	1.22^{ab}	0.04	1.33^{a}	0.03	1.23^{ab}	0.03	1.17^{b}	0.03	1.19^{b}	0.03	NS	**
GSI	21-27	0.14	0.02	0.16	0.02	0.13	0.01	0.18	0.02	0.18	0.02	NS	NS
DO%	21-25	87.1^{ab}	0.2	86.3^{a}	0.3	87.2^{ab}	0.2	87.7^{b}	0.3	87.0^{ab}	0.2	NS	**

Table S7. Condition factor (CF), liver index (HSI, %), gonadosomatic index (GSI, %) and dress-out percentage (DO%) in Atlantic in trial 2 fed dietary EPA+DHA of 1.3 to 7.4 % of total FA for 151days at 12°C. Mean values with SEM of individual fish per tank. Nested ANOVA did not show any significant differences in mean values per diet for any of the biometric indices.

	Fish/tank	Diet 2:1.3		Diet 2:2.7		Diet 2:4.4		Diet 2:7.4		Regression	Nested
	n	Mean	SEM	Mean	SEM	Mean	SEM	Mean	SEM	(*n* 8)	ANOVA
CF											
Tank 1	51-54	1.55	0.02	1.53	0.01	1.55	0.02	1.57	0.01		
Tank 2	50-58	1.57	0.02	1.56	0.02	1.54	0.02	1.61	0.02		
		1.56	0.01	1.54	0.01	1.55	0.01	1.59	0.01	NS	NS
HSI											
Tank 1	26-26	1.69	0.04	1.55	0.05	1.64	0.05	1.71	0.05		
Tank 2	25-28	1.69	0.05	1.64	0.07	1.82	0.05	1.73	0.05		
		1.69	0.03	1.60	0.04	1.74	0.04	1.72	0.03	NS	NS
GSI											
Tank 1	24-35	2.0	0.5	1.3	0.5	1.6	0.5	1.3	0.4		
Tank 2	27-35	1.4	0.5	2.1	0.5	1.7	0.5	1.8	0.6		
		1.7	0.4	1.7	0.3	1.6	0.4	1.5	0.3	NS	NS
DO%											
Tank 1	24-27	86.1	0.3	86.2	0.3	87.3	0.2	86.8	0.3		
Tank 2	19-26	86.3	0.3	87.0	0.2	86.0	0.3	87.6	0.2		
		86.2	0.2	86.6	0.2	86.2	0.2	87.1	0.2	NS	NS

Table S8. Content (mg g^{-1}) of moisture, protein, fat and ash in whole body of Atlantic salmon fed increasing levels of EPA+DHA at 12 and 6°C (trial 1) and at 12°C in trial 2. Values for pooled samples of 10 fish per tank in trial 1 and mean of 4 pooled samples of 5 fish each with SEM at start and of one pooled sample per tank at the end in trial 2. No significant regressions between proximate composition and diet.

	Diet	Moisture		Protein		Fat		Ash	
Trial 1									
Start	1:1.4	604		173		202		18	
	1:2.7	607		172		212		19	
	1:3.4	600		173		207		20	
	1:4.3	608		175		211		18	
	1:5.2	601		172		207		22	
End									
6°C	1:1.4	587		168		222		19	
	1:2.7	578		164		234		15	
	1:3.4	582		164		213		18	
	1:4.3	585		168		235		16	
	1:5.2	590		166		220		17	
12°C	1:1.4	602		168		218		18	
	1:2.7	597		168		217		18	
	1:3.4	591		171		223		19	
	1:4.3	589		169		224		20	
	1:5.2	597		161		214		20	
Trial 2									
			SEM		SEM		SEM		SEM
Start		624	3	178	1.6	179	3.8	20	1
End	2:1.3	603		161		220		19	
		599		157		235		16	
	2:2.7	593		157		245		17	
		582		162		249		20	
	2:4.4	593		159		226		21	
		584		158		248		16	
	2:7.4	588		159		243		16	
		588		162		245		16	

Table S9. Fatty acid composition (mg g^{-1}) in diets used in the temperature period in trial 1 and in trial 2.

Diet	1:1.4	1:2.7	1:3.4	1:4.3	1:5.2	2:1.3	2:2.7	2:4.4	2:7.4
Fatty acid									
14:0	2.4	4.6	6.5	7.5	9.2	1.8	3.4	5.0	7.1
16:0	54.6	55.3	60.5	56.3	56.7	44.8	45.6	44.5	42.6
18:0	8.2	8.2	8.6	8.3	8.2	8.2	8.3	8.4	8.1
Total saturates	*66.9*	*70.0*	*77.6*	*74.0*	*76.0*	*58.0*	*60.8*	*61.4*	*61.7*
16:1n-7	2.3	4.4	6.1	7.1	8.7	1.2	2.9	4.7	6.8
18:1n-9	144.1	133.6	139.1	128.9	116.3	125.9	121.2	111.4	95.2
20:1n-9	4.6	6.2	7.7	8.2	9.3	3.1	4.1	5.5	7.0
22:1n-11/9	2.7	4.9	6.7	7.7	9.3	2.1	3.6	5.6	8.2
Total monoenes	*164.8*	*164.0*	*176.7*	*162.7*	*159.9*	*139.0*	*139.5*	*135.2*	*125.4*
18:2n-6	59.2	56.0	58.2	52.3	49.2	46.9	44.1	40.6	34.0
20:4n-6	0	0	0	0.2	0.3	0.1	0.3	0.6	0.8
Total n-6	*53.1*	*49.3*	*46.9*	*44.9*	*44.7*	*47.2*	*44.6*	*41.7*	*35.4*
18:3n-3	46.8	43.0	44.8	38.7	36.4	36.2	33.3	31.4	25.4
18:4n-3	0.4	1.1	1.6	2.0	2.5	0.3	0.8	1.4	2.2
20:4n-3	0	0.3	0.4	0.5	0.6	0.1	0.2	0.3	0.5
20:5n-3	2.3	4.8	6.7	8.0	9.9	1.0	3.8	6.5	9.8
22:5n-3	0.3	0.7	0.9	1.1	1.3	0.1	0.4	0.7	1.1
22:6n-3	2.5	4.5	6.0	6.9	8.5	1.3	3.4	5.6	8.1
Total n-3	*52.3*	*54.6*	*60.6*	*57.5*	*59.6*	*39.5*	*42.6*	*46.8*	*48.3*

Table S10. Fatty acid composition (mg g^{-1}) in whole body of Atlantic salmon at the start and end of the temperature period in trial 1. Values for pooled samples of 10 fish per diet.

	Start 12 and 6 °C					Final at 12°C					Final at 6°C				
Diet	1:1.4	1:2.7	1:3.4	1:4.3	1:5.2	1:1.4	1:2.7	1:3.4	1:4.3	1:5.2	1:1.4	1:2.7	1:3.4	1:4.3	1:5.2
Fatty acid															
14:0	1.4	1.6	1.9	2.5	2.7	1.8	2.7	3.1	3.8	4.6	1.8	3.0	3.6	4.15	4.9
16:0	18.6	17.1	17.0	19.9	17.9	29.2	31.2	29.3	30.1	30.9	30.05	32.5	32.3	31.9	31.2
18:0	4.3	3.9	3.8	4.4	3.8	7.2	7.5	6.7	6.8	6.9	7.6	8.1	7.75	6.9	7.1
Total saturates	*24.8*	*23.1*	*23.2*	*27.4*	*25.0*	*39.2*	*42.4*	*40.1*	*41.8*	*43.5*	*40.3*	*44.5*	*44.5*	*43.8*	*44.2*
16:1*n*-7/9	1.6	1.8	2.2	2.8	3.0	2.2	3.3	3.4	4.3	5.1	2.65	4.2	4.55	5.35	5.8
18:1*n*-9/7	57.6	48.8	47.8	56.2	48.8	97.3	96.9	87.3	87.4	85.4	106.7	110.3	103.1	100.2	92.8
20:1*n*-9	2.8	2.7	3.1	3.7	3.6	5.2	6.3	6.3	6.9	7.6	5.45	6.75	7.35	7.85	7.9
22:1*n*-11/9	1.2	1.2	1.5	2.1	2.2	1.7	2.7	3.2	3.9	4.7	1.75	2.85	3.4	4.05	4.65
Total monoenes	*63.8*	*55.2*	*55.3*	*65.8*	*58.5*	*107.6*	*110.5*	*101.4*	*103.8*	*104.4*	*117.8*	*125.4*	*119.9*	*119.0*	*112.7*
18:2*n*-6	17.9	15.9	15.4	17.9	16.3	29.3	28.8	26.5	26.9	26.2	31.7	33.3	31.6	31.0	29.0
20:4*n*-6	2.0	3.6	2.9	4.7	2.6	0.4	0.4	0.4	0.4	0.4	0.5	0.4	0.4	0.5	0.5
Total n-6	*23.2*	*22.1*	*20.7*	*25.2*	*21.1*	*34.4*	*33.7*	*30.6*	*31.1*	*30.1*	*37.9*	*38.9*	*36.8*	*36.0*	*33.6*
18:3*n*-3	8.9	8.5	8.1	9.5	9.0	15.4	15.7	15.1	15.3	15.5	16	17.7	17.4	17.5	16.3
18:4*n*-3	3.0	1.9	1.8	2.1	1.6	4.0	3.1	2.7	2.7	2.5	4.6	3.9	3.1	2.7	2.7
20:4*n*-3	1.0	1.0	1.0	1.1	1.0	1.7	1.8	1.6	1.7	1.7	1.9	2.2	2.1	2.1	2.1
20:5*n*-3	1.7	1.7	1.8	2.4	2.6	2.4	2.8	2.8	3.3	3.9	2.5	3.2	3.4	3.9	4.3
22:5*n*-3	0.6	0.7	0.7	1.0	1.0	1.0	1.1	1.2	1.4	1.6	1.05	1.3	1.4	1.6	1.8
22:6*n*-3	4.3	4.4	5.1	6.3	6.5	4.5	5.6	6.2	7.4	8.4	4.6	5.7	6.5	7.8	8.9
Total n-3	*20.0*	*18.7*	*19.1*	*23.1*	*22.5*	*29.5*	*30.9*	*30.4*	*32.6*	*34.7*	*31.5*	*35.1*	*35.2*	*37.2*	*37.5*

Table S11. Fatty acid composition (mg g^{-1}) in whole body of Atlantic salmon at the start and end of trial 2. Values for pooled samples of 5 fish per tank and mean and SEM for four pooled samples of 5 fish from final and initial sampling, respectively.

	Initial		Final							
Diet			2:1.3		2:2.7		2:4.4		2:7.4	
		SEM	Tank1	Tank2	Tank1	Tank2	Tank1	Tank2	Tank1	Tank2
Fatty acid										
14:0	2.36	0.06	1.83	1.96	2.73	2.77	3.31	3.66	5.26	5.35
16:0	22.85	0.65	27.86	30.23	31.61	32.56	30.25	33.11	31.78	31.81
18:0	5.60	0.24	6.53	7.05	7.19	7.41	7.08	7.51	7.13	7.26
Total saturates	*32.08*	*0.98*	*37.6*	*40.7*	*43.1*	*44.3*	*42.2*	*45.9*	*45.9*	*46.2*
16:1*n*-7/9	3.0	0.06	2.46	2.68	3.54	3.64	4.06	4.52	5.64	5.79
18:1*n*-9/7	73.07	2.05	91.86	98.86	99.53	101.59	90.41	97.481	86.18	87.32
20:1*n*-9	4.44	0.15	4.74	5.04	5.95	6.09	6.18	6.78	7.35	7.39
22:1*n*-11/9	2.48	0.02	2.11	2.22	3.00	2.96	3.63	3.90	5.66	5.80
Total monoenes	*83.85*	*2.46*	*102*	*110*	*113*	*115*	*105*	*114*	*106*	*108*
18:2*n*-6	23.61	0.63	28.55	30.83	31.35	31.80	28.45	30.47	27.82	28.09
20:4*n*-6	1.16	0.05	1.24	1.32	1.56	1.60	1.58	1.76	1.80	1.80
Total n-6	*27.95*	*0.79*	*34.2*	*36.6*	*37.3*	*37.8*	*33.7*	*36.3*	*33.2*	*33.5*
18:3*n*-3	12.35	0.38	14.60	16.33	17.07	17.60	16.11	17.17	16.19	16.23
18:4*n*-3	2.07	0.04	3.00	3.17	2.77	2.80	2.17	2.42	2.36	2.45
20:4*n*-3	1.45	0.08	1.85	1.73	1.96	1.98	1.84	1.90	2.13	2.17
20:5*n*-3	2.30	0.07	2.07	2.34	2.82	2.95	3.16	3.41	4.84	4.62
22:5*n*-3	0.88	0.04	0.81	0.84	1.11	1.13	1.25	1.42	2.09	2.03
22:6*n*-3	4.45	0.09	3.73	3.32	4.87	4.82	5.93	6.28	8.11	8.60
Total n-3	*24.20*	*0.70*	*26.7*	*28.4*	*31.4*	*32.0*	*31.3*	*33.6*	*36.8*	*37.3*

Table S12. Fatty acid composition (% of FA) in fillet of Atlantic salmon at the start and end of the temperature period in trial 1. Mean values for 6 and 8 fish per tank at start and end, respectively.

	Start 12 and 6 °C					Final at 12°C					Final at 6°C				
Diet	1:1.4	1:2.7	1:3.4	1:4.3	1:5.2	1:1.4	1:2.7	1:3.4	1:4.3	1:5.2	1:1.4	1:2.7	1:3.4	1:4.3	1:5.2
Fatty acid															
14:0	1.0	1.3	1.5	1.7	2.0	0.7	1.1	1.3	1.6	1.9	0.7	1.1	1.4	1.6	1.9
16:0	13.2	13.6	13.5	13.4	13.1	12.5	12.9	13.1	12.9	13.2	12.1	12.3	12.5	12.4	12.5
18:0	3.1	3.2	3.0	3.0	2.8	3.2	3.1	3.1	3.0	3.0	3.1	3.1	3.1	2.7	2.9
Total saturates	*17.8*	*18.7*	*18.6*	*18.8*	*18.7*	*17.1*	*18.0*	*18.4*	*18.4*	*19.0*	*16.6*	*17.1*	*17.7*	*17.4*	*18.1*
16:1*n*-7/9	1.2	1.5	1.8	1.9	2.2	1.0	1.4	1.6	1.8	2.2	1.1	1.6	1.8	2.1	2.4
18:1*n*-9/7	41.8	39.5	38.7	38.7	36.6	42.8	41.1	40.3	38.6	37.3	44.1	42.7	41.1	39.9	38.2
20:1*n*-9	2.1	2.2	2.5	2.6	2.7	2.3	2.7	2.9	3.1	3.3	2.3	2.7	3.0	3.2	3.3
22:1*n*-11/9	0.9	1.1	1.3	1.5	1.7	0.8	1.2	1.5	1.7	2.1	0.7	1.1	1.4	1.6	2.0
Total monoenes	*46.4*	*44.8*	*44.7*	*45.3*	*43.9*	*47.3*	*46.9*	*46.7*	*45.9*	*45.7*	*48.7*	*48.5*	*47.8*	*47.3*	*46.5*
18:2*n*-6	13.1	13.0	12.5	12.4	12.3	12.9	12.3	12.3	11.9	11.5	13.2	13.0	12.7	12.5	12.0
20:4*n*-6	0.4	0.3	0.3	0.3	0.3	0.2	0.2	0.2	0.2	0.2	0.3	0.2	0.2	0.2	0.2
Total n-6	*15.9*	*15.4*	*14.8*	*14.4*	*14.3*	*15.2*	*14.5*	*14.2*	*13.7*	*13.2*	*15.9*	*15.2*	*14.8*	*14.5*	*14.0*
18:3*n*-3	6.6	7.1	6.8	6.6	6.9	6.9	6.8	7.0	6.8	6.9	6.7	6.9	7.0	7.1	6.8
18:4*n*-3	2.2	1.6	1.5	1.5	1.3	1.8	1.3	1.3	1.2	1.1	2.0	1.5	1.3	1.1	1.1
20:4*n*-3	0.8	0.8	0.8	0.8	0.8	0.8	0.8	0.7	0.8	0.8	0.9	0.9	0.9	0.9	0.9
20:5*n*-3	1.3	1.5	1.5	1.7	2.1	1.0	1.2	1.3	1.4	1.7	1.1	1.3	1.4	1.7	1.9
22:5*n*-3	0.5	0.6	0.6	0.7	0.8	0.4	0.5	0.5	0.6	0.7	0.5	0.6	0.6	0.7	0.8
22:6*n*-3	3.4	3.9	4.5	4.6	5.2	2.1	2.5	3.0	3.4	3.9	2.0	2.3	2.7	3.3	3.9
Total n-3	*15.1*	*15.9*	*16.1*	*16.4*	*17.5*	*13.2*	*13.5*	*14.3*	*14.7*	*15.6*	*13.3*	*13.9*	*14.4*	*15.3*	*15.9*

Table S13. Fatty acid composition (% of total FA) in fillet of Atlantic salmon at the start and end of trial 2. Mean values for 16 and 10 fish per tank at start and end, respectively.

	Initial	Final							
Diet		2:1.3		2:2.7		2:4.4		2:7.4	
		Tank1	Tank2	Tank1	Tank2	Tank1	Tank2	Tank1	Tank2
Fatty acid									
14:0	1.3	0.9	0.8	1.1	1.1	1.4	1.4	2.1	2.2
16:0	12.7	12.7	12.9	12.6	12.8	13.0	12.9	12.8	13.2
18:0	2.9	2.7	2.8	2.8	2.9	2.8	2.8	2.8	2.8
Total saturates	*17.8*	*17.0*	*17.2*	*17.3*	*17.5*	*18.2*	*18.0*	*18.6*	*19.2*
16:1*n*-7/9	1.6	1.0	1.1	1.3	1.4	1.6	1.7	2.3	2.4
18:1*n*-9/7	39.6	41.1	41.0	40.1	40.0	37.9	38.2	34.8	34.9
20:1*n*-9	2.6	2,4	2,4	2,5	2,6	2,8	2,9	3,3	2,8
22:1*n*-11/9	1.3	0.6	0.9	1.2	1.2	1.5	1.5	2.3	2.2
Total monoenes	*45.8*	*45.8*	*46.0*	*45.7*	*45.8*	*44.6*	*44.8*	*43.5*	*43.4*
18:2*n*-6	13.5	13.6	13.7	13.5	13.3	12.6	12.7	11.8	11.7
20:4*n*-6	0.9	0.2	0.2	0.2	0.2	0.2	0.2	0.2	0.2
Total n-6	*15.8*	*16.1*	*16.1*	*15.7*	*15.4*	*14.7*	*14.7*	*13.8*	*13.6*
18:3*n*-3	7.5	7.2	7.5	7.6	7.4	7.2	7.3	7.0	7.2
18:4*n*-3	1.4	1.6	1.5	1.3	1.2	1.2	1.2	1.1	1.1
20:4*n*-3	0.9	0.8	0.8	0.8	0.8	0.8	0.8	0.8	0.8
20:5*n*-3	1.6	1.3	1.3	1.5	1.5	1.7	1.7	2.3	2.3
22:5*n*-3	0.6	0.4	0.5	0.5	0.6	0.6	0.6	0.9	0.9
22:6*n*-3	3.2	2.2	2.4	2.6	2.6	3.5	3.6	4.3	4.4
Total n-3	*15.6*	*14.0*	*14.4*	*14.9*	*14.8*	*15.7*	*15.7*	*17.1*	*17.3*

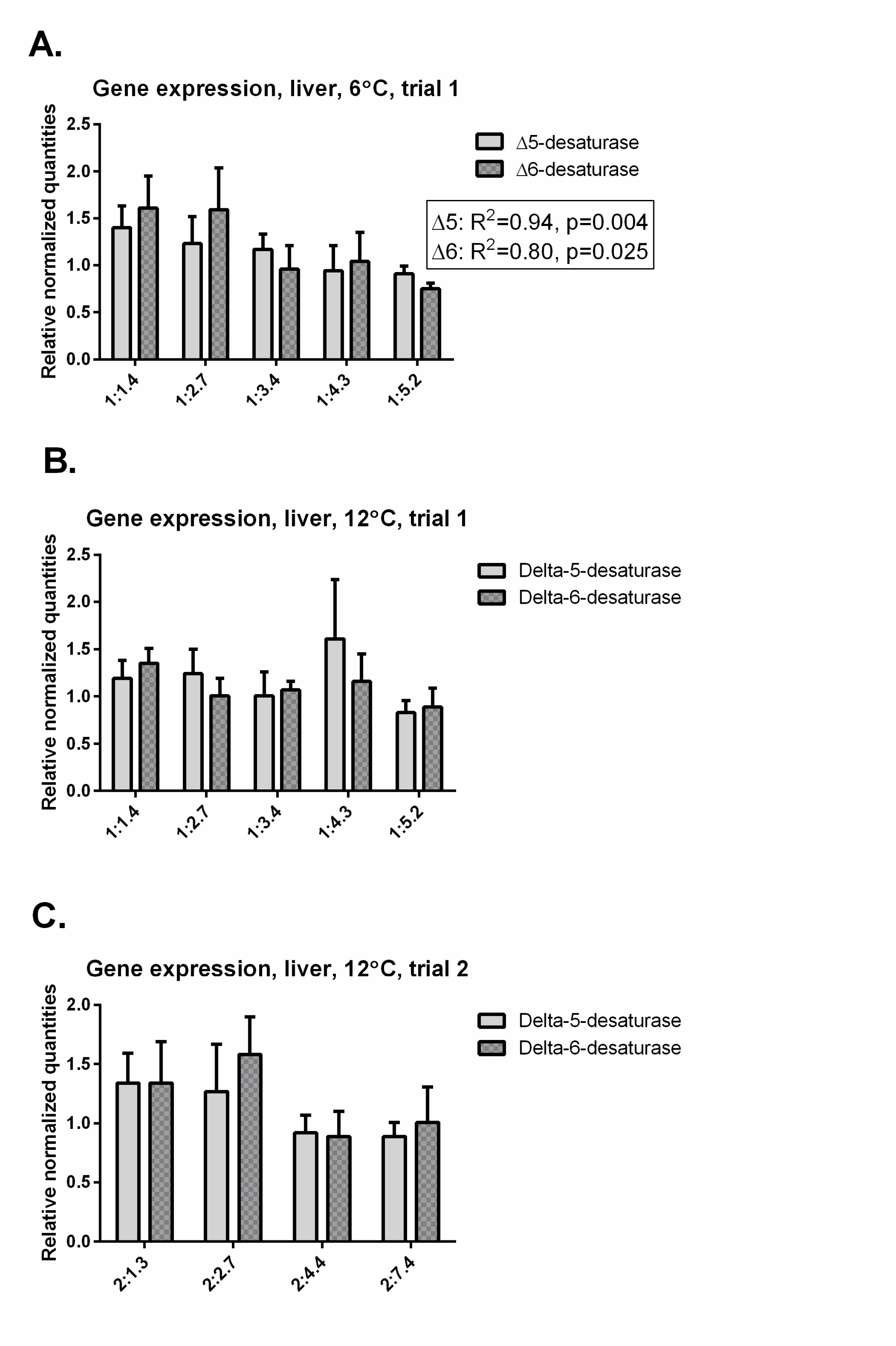

Fig. S1. Normalized expression of the genes Δ5- and Δ6-desaturase in liver tissue from Atlantic salmon fed dietary levels from 1.4 to 5.2 % of total FA for 202 and 142 days at 6 and 12°C, respectively (A and B) and 1.3 to 7.4 % of total FA for 151 days at 12°C (C). Significant linear regressions are shown in the figures. The bars represent the mean, while the error bars represent SEM.
